# Digital Identity: The effect of trust and reputation information on user judgement in the Sharing Economy

**DOI:** 10.1371/journal.pone.0209071

**Published:** 2018-12-13

**Authors:** Mircea Zloteanu, Nigel Harvey, David Tuckett, Giacomo Livan

**Affiliations:** 1 Department of Computer Science, Faculty of Engineering, University College London, London, United Kingdom; 2 Department of Experimental Psychology, Division of Psychology and Language Sciences, University College London, London, United Kingdom; 3 Centre for the Study of Decision-Making Uncertainty, University College London, London, United Kingdom; 4 Systemic Risk Centre, London School of Economics and Political Sciences, London, United Kingdom; Baylor University, UNITED STATES

## Abstract

The Sharing Economy (SE) is a growing ecosystem focusing on peer-to-peer enterprise. In the SE the information available to assist individuals (users) in making decisions focuses predominantly on community-generated trust and reputation information. However, how such information impacts user judgement is still being understood. To explore such effects, we constructed an artificial SE accommodation platform where we varied the elements related to hosts’ digital identity, measuring users’ perceptions and decisions to interact. Across three studies, we find that trust and reputation information increases not only the users’ perceived trustworthiness, credibility, and sociability of hosts, but also the propensity to rent a private room in their home. This effect is seen when providing users both with complete profiles and profiles with partial user-selected information. Closer investigations reveal that three elements relating to the host’s digital identity are sufficient to produce such positive perceptions and increased rental decisions, regardless of which three elements are presented. Our findings have relevant implications for human judgment and privacy in the SE, and question its current culture of ever increasing information-sharing.

## Introduction

### Background

The Sharing Economy (SE) describes a growing ecosystem of online platforms devoted to the exchange of goods and services [1–4]. While a precise and encompassing definition of “sharing economy” is still debated within academia and business [2,5,6], the concept is grounded in peer-to-peer (P2P) enterprise, providing individuals with temporary access to the resources of other individuals [7], while using the platforms simply as an exchange mediator [3,8]. In this respect, despite sharing its peer-to-peer nature with other online environments (such as e-commerce websites), the SE represents a distinctly unique concept.

The SE has seen fantastic growth and high adoption rates among the general population [5], creating value on an unprecedented scale [3,9], and rivaling well established sectors of the traditional economy [10]. The types of platforms that have emerged around the SE paradigm range across all domains and markets, from accommodation (e.g., Airbnb) and taxi services (e.g., Uber) to household appliances (e.g., Zilok) and clothes (e.g., GirlMeetsDress).

### Trust and reputation

In most marketplaces, individuals are aware that the services they request are subject to regulations, consumer protection laws, and monitoring by governmental bodies, ensuring a degree of liability, security, and safety. Within the SE, such protections are reduced [5], as SE platforms offer direct and largely unmediated interaction between individuals who have never met before neither offline nor online [1,3]. SE operations require a high level of trust on behalf of all parties involved [11], which needs to be established on a personal level that goes well beyond the mere exchange of goods, in the absence of indicators typically employed to signal quality or reliability in traditional markets. In this respect, trust in the SE differs significantly from the trust associated to other forms of online economic exchange, such as business-to-consumer and consumer-to-consumer e-commerce [2,6,12].

In recent years, the role of trust in the online environment has received significant attention [13–19]. While its precise definitions may vary depending on the context, here trust refers to the psychological state reflecting the willingness of an actor to place themselves in a vulnerable situation with respect to the actions/intentions of another actor, in the absence of a direct ability to monitor or control the other party [20,21]. Thus, trust always involves some aspect of risk, uncertainty, vulnerability, and the expectation of reciprocation.

By extension, the ability to infer the trustworthiness of the individuals with whom users wish to engage is fundamental to the SE’s operation [11,22–26]. This process is typically facilitated by requiring SE users to establish a digital identity (DI; namely, what the user decides to share on the platform and the information generated by the community, see the next Section). Hence, following Todorov and colleagues [27,28], throughout this paper we operationalize trust in the SE in terms of the *perceived trustworthiness* of its users, and how this is affected by the information they share on SE platforms. For the sake of brevity, throughout the paper our discussions regarding the roles and effects of trust refer to this concept of trustworthiness.

Individuals in the SE (i.e. users), themselves, gravitate towards information relating to the trustworthiness of the individuals they wish to engage with before deciding to interact [29–31]. Furthermore, research has shown that trust, in turn, has a significant impact on user decision-making and behavior [13,15,17,32].

Another component driving user behavior within the SE is user reputation. Reputation in the SE reflects the perception the community has towards a user, through their longevity on a platform, contributions to the community, and outcome of past engagements (typically, represented as numeric or valence scores). Reputation can be considered an aggregate representation of trust towards a certain individual or entity. Thus, in the SE a good reputation can effectively perform a regulatory role conducive to trust [25,31]. All in all, trust and reputation can be considered the most valuable commodities within the SE [3,33,34].

Trust and reputation information are relevant due to the implicit information asymmetry and economic risks of SE platforms, forcing users to rely on such elements to inform their decision-making [29,33,35,36]. The success of such a market relies on its ability to reduce uncertainty in its P2P interactions [3]. In such markets, often, the only source that allow people to infer the credibility of another user is to relying on their digital identify information. Thus, trust is crucial for turning a user’s uncertainty into a definitive request to use a service [22].

### Digital identity

DI is often used as a blanket term encompassing the overall online footprint of a given entity, be it an individual or a company. While in general DI is undoubtedly a complex and multifaceted concept, in the SE setting it acquires a rather precise meaning, which arises from the interplay of the information SE users willingly share and the information their peers share about their past interactions with them.

At the core of any SE platform are systems that provide *reputation* building information [37], which typically aggregate *subjective* user generated content (UGC) into a reputation score; this in turn forms the core of a user’s DI on the platform. Most SE platforms actively promote mechanisms through which users can *share* information, *rate* others, and build a *reputation* on the platform. Such content usually takes the form of numerical (e.g., ratings between 1 and 5) and text reviews. Each user has to carefully consider the reputation they foster within the community, ensuring that their identity convinces others that they are trustworthy and would want to interact with them.

Depending on the platform, the above information is typically complemented with more *objective* data through which users can signal trust and build relationships [38]. These include, but are not limited to, identity verification, photos of sellers and their goods, and sellers’ contact information [39]. The combination of these two types of systems acts to reduce the implicit uncertainty of operating in such markets [20,23,40]. Yet, still little is known about how SE users incorporate such information into their decision-making processes.

## Aims & research questions

The central research question we address is whether DI impacts the perceived trustworthiness of SE users. In particular, our goal is to investigate whether SE users are able to integrate the wealth of available information on their peers’ profiles when deciding with whom to interact. Our initial hypothesis is twofold. On one hand, we hypothesized that SE users are strongly affected by the presence of trust and reputation information (TRI), leading to changes in their judgements towards hosts and the services they offer, possibly without even realizing the strength of such effects. Yet, the literature on decision making [41–44] informs us that people often are incapable of integrating information from multiple sources, and unwittingly make decisions based on a limited number of cues (typically two or three). We expect the same phenomenon to take place in the SE as well, i.e., we hypothesized that SE users’ judgments are unaffected by the increase in the available information shown on their peers’ profiles above a certain amount.

We tested these hypotheses by designing an artificial accommodation platform, which allowed us complete control over the type and amount of information displayed to users. Hypothetical decision-making scenarios allow the experimental investigation of actual decision-making processes, and findings from them have been generally found to be consistent with field-collected data [45]. The profiles were generated to resemble accommodation SE platforms (e.g., Airbnb). Our choice was motivated by the rise of the SE being historically driven by a handful of companies, Airbnb being the most globally prominent of them. In this respect, our choice facilitated ecological validity and the participants’ awareness of the experimental context. Over three experiments, individuals (users) were presented with profiles reflecting hosts wishing to rent a private room in their house. The aim was to understand whether the TRI available on hosts’ profiles has an effect on users’ decisions to rent a private room advertised by a host.

Our first study was exploratory and aimed at collecting data of SE users’ choice patterns. Indeed, Study 1 assessed the effect of providing users with different amounts of host related TRI. This contrasted host profiles containing minimal information (akin to those of new users on a platform), with profiles containing the full information typically seen on such sites, and with profiles where the information presented was user-selected (allowing for some understanding of the users’ underlying thought processes).

The results from Study 1 contributed to corroborate the aforementioned hypotheses, which were investigated more deeply in the two following studies. Study 2 focused on the importance of selecting versus being given the information of interest. Finally, in Study 3, the importance of the type of information users see was addressed. Here, the aim was to uncover whether specific TRI elements impact user judgements.

Across all three studies several dependent variables were measured. Initially, the participants provided their rent decision for the room advertised, then rated their confidence in this judgment. Subsequently, they provided ratings for their perceived trustworthiness, credibility, and sociability of the hosts advertising each room. This was done in order to assess how differences in the amount and type of TRI impacted these dimensions. Specifically, we explored how providing users such information would affect how trustworthy a host appeared, whether having TRI on the platform would make the information hosts provided seem more credible, and whether providing user-generated content from past interactions would make the hosts appear more sociable. This choice was made as SE platforms often brand themselves as environments that favor personal interactions in a unique way. Therefore, in our experiments we introduced TRI elements that, although not always present in real-world SE platforms, were designed to test differences in those dimensions (see Study 1‘s Stimuli Section).

## Study 1

The first study investigated the effect of providing users with different amounts of host-related TRI, over three conditions: Hidden, Visible, and Reveal. This contrasted host profiles lacking TRI (Hidden), with full profiles (Visible), and profiles where only partial user-selected information was presented (Reveal). The effect of differences in the amount of TRI on user judgments was measured on ratings of host credibility, trust, sociability, rent decisions, and confidence. It was predicted that the increased amount of information available would impact users’ perceptions of hosts, resulting in differences in ratings, and decision to rent the private rooms. Additionally, allowing users to select host TRI would reveal which elements facilitate decision-making on SE platforms, or at least which elements users believe facilitates their decisions. The utility of the Reveal condition was in observing participant choices directly, preventing various participant response biases, such as responding according to or against what they surmise the experiment’s goal to be [46].

### Methods

#### Participants and design

A total of 160 participants were recruited online through Amazon’s Mechanical Turk (mTurk; www.mturk.com) in exchange for a flat fee of $1.00. After deleting incomplete or invalid cases (*n* = 36) the final data encompassed 124 participants.

Eiteee asd (65 males, 58 females, one undisclosed; *M*_*Age*_ = 35.11, *SD* = 10.52; range: 20–73). Written (digital) informed consent was obtained from all participants prior to participation. This study and all procedures used have been ethically reviewed and received ethics approval from the University College London Research Ethics Committee (CEHP/2015/534).

An independent-samples design was used, with three levels of Profile (Hidden, Visible, and Reveal). Participants were measured on multiple dependent variables: rent decision, confidence in decision, perceived sociability of host, trustworthiness of the host, and credibility of information (also, see **S1 Text**).

#### Stimuli

**Fig 1:**
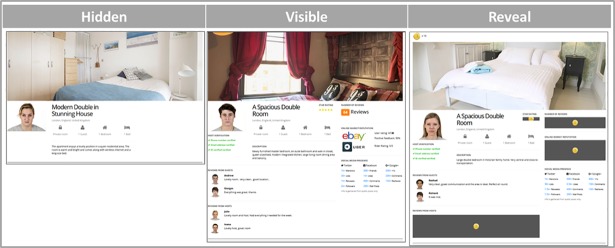
Example of host profiles in each condition, indicating which elements were visible to users. In the Hidden condition, only a picture of the host, one of the room, and a minimal description of the latter were present (NB: these elements were visible in all conditions). In the Visible condition, 7 additional elements were shown to users. In the Reveal condition, users had to spend tokens to visualize any 3 out of such 7 elements (the profile above already shows the 3 users-selected elements, with the 4 non-selected elements remaining obscured).

Typical SE accommodation profiles were created specifically for the purposes of this experiment, using the Gorilla platform (www.gorilla.sc). These contained the elements generally featured on such sites, with the addition of two elements. The profiles were described as representing a “private room” in the host’s house that they wished to rent out to potential guests (see Fig 1).

The elements were as follows: a photo of the advertised room, a description of the room, a photo of the host, host verification, two guest reviews, two host reviews, online market reputation, social media presence, number of reviews, and star rating (for examples, see **S2 Text**).

Certain factors were controlled in the creation of the profiles. To ensure quality and ecological validity, all elements were created to reflect the general ratings and content observed on SE platform profiles [4,47]. Two new elements were uniquely created to introduce further digital identity cues about the hosts: “social media presence” and “online market reputation”. This decision stems from our perception of SE platform trends towards increased information-sharing and the role that users’ DI has on their online perception [30].

The former element was introduced, in particular, to gauge its impact on perceived host sociability (as well as all other trust-related metrics, see **S2 Text**). Indeed, SE platforms often market themselves as environments that favor unique personal interactions, which motivated our choice to introduce an additional TRI element to test sociability.

The latter element, instead, was introduced to assess how cross-platform user reputation impacts decision-making on other platforms (i.e. the reputation of the user on one P2P platform influencing how they are perceived on another P2P platform). Including this type of information has been advocated by many, both in the industry and academia (cf. [48]), and in recent years several startup companies have put forward solutions in this direction. For a comprehensive description of element creation and controls implemented see **S2 Text**.

#### Procedure

Participants were randomly assigned to one of the three Profile conditions; Hidden (*n* = 42), Visible (*n* = 40), and Reveal (*n* = 42). They were first given a series of pre-task questions, including demographics (age, gender, and ethnicity), and SE usage (see **S1 Text**). They then received instructions specific to their condition and were provided with an example profile, familiarizing them with the layout of the profiles, the type of information available, and the responses they would need to provide.

During the main task, users saw one profile at a time, and were asked if they wanted to “Rent” or “Not Rent” (binary, forced-choice). The other DV questions were measured on a 10-point Likert-type scale. Confidence was phrased as “How confident are you in your decision to Rent/Not rent?” (1—*Not at all Confident* to 10—*Very Confident*). Sociability was phrased as “How sociable do you think the host would be?” (1—*Not at all Sociable* to 10—*Very Sociable*). Trustworthiness was phrased as “How would you rate the trustworthiness of the host?” (1—*Very Untrustworthy* to 10—*Very Trustworthy*). While credibility was phrased as “How credible do you think the information about this room is?” (1—*Not at all Credible* to 10—*Very Credible*).

This was repeated over 10 trials. After each trial they were given a “validation” question, to ensure they were paying attention to the information in the profile. This was in the form of a question about a specific element on the profile (e.g., “What were the colors of the walls?”, with an open-ended response). Users who attempted to go back in order to check profiles once more before answering the validation question were treated as invalid cases and discarded from the analysis. The elements comprising each profile were randomized between participants, reducing the artificial influence of specific combination on the responses. The inclusion of the validation questions in our design was warranted due to the attention benefits such questions can have on respondents (see [49]). In this respect, our design was in line with well-established practices for web-based experiments [49]. However, we note that data on user accuracy on the validation questions was not collected, and thus we cannot make inferences as to their relationship with user judgements.

In the Hidden condition, users saw a host’s profile with minimal information presented about a room offered. This was limited to a photo of the room, a picture of the host, and a description relating to the room; these were always shown regardless of the profile condition. In the Visible condition, users saw a fully populated host profile, containing all the elements detailed above. In the Reveal condition, for each profile users had three tokens to “spend” on revealing any information they desired to help in their judgements. The elements available were: host verification, guest reviews, host reviews, online market reputation, social media presence, number of reviews, and star rating. The information regarding the spending of the three tokens was recorded in each trial. After the main task was completed, participants had to answer several post-task questions, and were debriefed (see **S1 Text**).

### Results

Users’ ratings were summed across the 10 trials and analyzed on each of the five dependent measures based on the three Profile conditions.

For decisions to rent the rooms, a one-way analysis of variance (ANOVA) revealed a significant main effect of Profile, *F*(2,121) = 3.44, *p* = .035, η^2^ = .054. Post-hoc Tukey’s HSD tests showed a significantly higher number of rent decisions in the Reveal condition (*M* = 8.05, *SD* = 2.23) than the Hidden condition (*M* = 6.60, *SD* = 2.43) and, *p* = .027. No other comparisons were significant.

Confidence in rent decisions was not found to be affected by Profile condition, *F* < 1, *p* = .893, suggesting that the type and amount of information participants saw on the profiles did not influence their confidence. Overall, the average confidence was very high for rent decisions (*M* = 7.58, *SD* = 1.3 per profile).

Ratings of host sociability were significantly affected by Profile condition, *F*(2,121) = 6.41, *p* = .002 , η^2^ = .096. Post-hoc tests showed significant lower user ratings for the Hidden condition (*M* = 60.81, *SD* = 16.07) compared to both the Visible (*M* = 70.83, *SD* = 13.69), *p* = .006, and the Reveal conditions (*M* = 70.50, *SD* = 13.56), *p* = .008.

Similarly, a main effect of host trustworthiness based on Profile was found, *F*(2,121) = 8.94, *p* < .001 , η^2^ = .129. Post-hoc tests revealed significantly lower ratings in the Hidden condition (*M* = 63.50, *SD* = 15.50) compared to both the Visible (*M* = 75.13, *SD* = 14.29), *p* = .001, and the Reveal (*M* = 74.36, *SD* = 12.07) conditions, *p* = .002. No other significant comparisons were found.

Finally, a main effect of percevied credibility was found, *F*(2,121) = 7.00, *p* < .001 , η^2^ = .104. Post-hoc comparisons revealed that the Hidden condition (*M* = 67.48, *SD* = 14.64) produced significantly lower ratings than both the Reveal (*M* = 76.69, *SD* = 11.87), *p* = .005, and Visible (*M* = 77.05, *SD* = 13.04), *p* = .004, conditions.

As the data suggest a lack of difference in user ratings between the Visible and Reveal condition, Bayesian independent-samples t-tests were conducted to complement the frequentist analysis. Considering the null hypothesis of no difference between the two conditions, tentative results were found in support of this claim. For all measures, the Bayes factor equaled between BF_01_ = 2.6–4.34, indicating that the data was around 3 to 4 times more likely under then null than the alternative hypothesis (**S3 Text**).

#### Triplet analysis

To obtain a better understanding of user selection preference for elements in SE platforms, an analysis of user token “spending” patterns in the Reveal condition was conducted. Each trial from each participant was treated as a unique vector of element selection from the total seven available items. Thus, the triplets of each of the 42 participants over the 10 trials were analyzed, 420 triplets in total. The triplet selection patterns were compared to a null model of random selection, using a binomial distribution (for details, see **S4 Text**).

Initially, with respect to the frequency of observation of certain elements, a few results are relevant. It was found that 89.5% of the selected triplets featured *at least* one of the two following elements: “star ratings” and “guest reviews”. Furthermore, 47.1% of the triplets featured *both* items, suggesting a strong user preference towards the two items. When testing for the over-representation of triplets, we find three combinations to be statistically significant at the 1% univariate level. These are: “star ratings + guest reviews + number of reviews”, “star ratings + guest reviews + host verification”, or “star ratings + guest reviews + host reviews”. These were interpreted as the triplets of TRI information users prefer most to aid in their decision-making.

### Discussion

This study finds that providing users with typical SE platform TRI increases their positivity towards their peers, rating them higher on sociability, trustworthiness, and credibility. Importantly, this also resulted in an increased number of rent decisions. Differences were observed when comparing user ratings in the Hidden condition, where all but the basic information was present, with the Visible and Reveal condition, where reputation and trust information was provided to varying degrees. The results also suggest that seeing a full profile (Visible) or one with only partial, but user-selected, information (Reveal) results, on average, in similar judgements towards hosts. Considering the difference in information between the two conditions (i.e. seven elements in the Visible condition and three user-selected elements in the Reveal condition), two explanations are proposed.

Potentially, the act of selecting which elements to see impacted users’ perception, leading to the belief that these elements are the most relevant/useful for their decision [50]. Thus, the act of selecting specific information may be generating this “positivity effect” (i.e. increased ratings towards hosts on all measures).

Alternatively, it may be that users rely only on a few elements when making their decisions, as argued by past decision-making research [50–52]. Thus, users may not be able to incorporate in their judgment the additional information present in a full profile.

A final noteworthy finding is that confidence in user decision was generally high and was not affected by the Profile condition. This suggests that the TRI is not a factor that impacts how confident users are in their SE rental decisions.

## Study 2

Study 2 was devised to unpack the effects relating to the act of selection and the amount of information on user judgement, as the data indicated that users relying on three selected elements rated hosts similarly to participants which had access to all seven elements.

People often report using complex strategies and a large number of cues when making their judgements; however, empirical studies show that they fail to accurately use more than two or three cues at any one time, and incorrectly predict which ones play a role in their decision-making process [41,43,44,50,52]. Thus, the lack of difference due to the amount of information may be explained either by the fact that (1) users considered the elements they selected as being more informative towards their final decision, leading to equally positive judgements, or (2) users seeing more than three elements could not incorporate these into their decision-making (i.e. discounting information), thus producing no differences in judgement.

A modification of Study 1’s design was implemented. Using the results from the Reveal condition, we selected element triplets to present to users directly and compared their responses to users that selected three elements. This ensured that the amount of information was kept constant (i.e. three pieces of TRI per profile), and that any difference in renting decision or perceptions of hosts would be a result of the act of selecting information.

### Methods

#### Participants and design

120 participants were recruited through mTurk, in exchange for $1.00. After deleting incomplete cases (*n* = 3), 117 participants remained.

Eiteee asd (65 males, 52 females; *M*_Age_ = 36.21, *SD* = 10.35; range: 20–69). The design was similar to that of Study 1, with Profile having two conditions: 3-Seen, 3-Reveal. Participants were measured on the same DVs as Study 1.

#### Stimuli

The profiles in the 3-Seen condition contained only three TRI elements to assist in user decision-making, selected based on the data from the previous study. In this condition each profile contained one of the three triplet combinations whose selection frequency was found to be significant in Study 1: “stars + guest reviews + number of reviews”, “stars + guest reviews + host verification”, or “stars + guest reviews + host reviews”. In the 3-Reveal condition, the same profile generation procedure as in Study 1’s Reveal condition was employed. This allowed for a direct comparison of the effect of choice on user judgement, as information was kept constant in all conditions.

#### Procedure

The procedure was identical to that of Study 1, but here participants were randomly assigned to one of the two Profile conditions: 3-Seen (*n* = 61) or 3-Reveal (*n* = 56). Within the 3-Seen condition, participants were further randomly divided into the three sub-conditions, based on the triplet they saw. Thus, each participant in the 3-Seen sub-condition saw a specific triplet throughout his/her trials (randomized between participants with a rate of 1/3). In all conditions participants saw 10 profiles and responded to questions regarding each profile using the same scales as Study 1. A control was also placed on the experiment which prevented participants from double-checking profiles after seeing the validation questions.

### Results

Multiple independent samples t-tests were conducted to compare the responses in the 3-Seen and 3-Reveal conditions for each DV. The data revealed no significant differences in any of the responses users gave based on Profile condition, on any of the measured variables, *t*s ≤ 1, *p*s > .05. This supports the explanation that users do not rely on more than three pieces of information judging a host’s profile (for additional analyses, see **S5 Text**).

To provide more support, Bayesian independent-samples t-tests were conducted on the data, which did favor this conclusion. Moderate to substantial support in favor of accepting the null of no difference between the 3-Seen and 3-Reveal conditions was found, BF_01_ between 2.29 and 3.77 for all DVs (**S5 Text**).

### Discussion

The data suggests that users are not affected by the act of selecting which TRI they want to see before making a rental decision beyond simply being presented three pieces of information. On all measured variables, users provided similar responses in the 3-Seen and 3-Reveal conditions. This suggests that users’ judgements are influenced by seeing at least three elements of TRI, but are unaffected by either the act of selecting said information or seeing more than three (**S5 Text**).

## Study 3

People find uncertainty unpleasant and seek to reduce it [53]. Relying on information provided by others may assist with this issue, allowing users to reduce the number of alternatives and task complexity [54]. However, the results from the above studies raise questions about the actual usefulness to users of the information available on SE platforms.

Is the information in the elements presented to users impacting decisions, or do users simply need three elements to believe they are making an “informed” decision? To understand this, the third study assessed whether *specific* triplets result in differences in users’ judgements about hosts’ credibility, sociability, trustworthiness, and decision to rent a room. Here, users saw one of three options. From Study 1’s Reveal condition, specific triplets were chosen to reflect the least selected triplet combinations (3-Avoided), the three most selected triplet combinations (3-Wanted), and triplets containing a random combination of the remaining elements (3-Random).

A significant difference in users’ judgements between conditions would support the claim that users have a preference for what information they use when making their rental decisions, which in turn affect their ratings of hosts. Alternatively, a lack of a difference would suggest that users simply require the presence of three elements for the positivity effect to occur.

### Methods

#### Participants and design

189 participants (103 males, 86 females; *M*_*Age*_ = 34.09, *SD* = 10.23; range: 19–48) were recruited through mTurk, in exchange for $1.00. The design was similar to the previous studies, with Profile having three conditions: 3-Wanted, 3-Avoided, 3-Random. Participants were measured on the same DVs as the previous studies.

#### Stimuli

In the 3-Wanted condition, profiles were generated using the same three triplet combinations as in Study 2’s 3-Seen condition. In the 3-Avoided condition, profiles were comprised of three triplet combinations chosen so as to be the least selected combinations by users in Study 1’s Reveal condition (see **S4 Text**). In these sub-conditions, “host verification” and “host reviews” were always shown together, while the third element was either “social media presence”, “online market reputation”, or “number of reviews”. In the 3-Random condition, profiles were comprised of randomly selected triplet combinations formed from the remaining unused combinations.

#### Procedure

Participants were randomly allocated to one of the three Profile conditions: 3-Wanted (*n* = 67), 3-Avoided (*n* = 67), 3-Random (*n* = 55). For the 3-Wanted and 3-Avoided conditions, participants were further divided into their respective three sub-conditions. Participants saw 10 host profiles in each condition containing a consistent triplet of information throughout. They responded to each profile using the same scales as in the previous studies.

### Results

Multiple one-way ANOVAs were conducted to uncover any differences in users’ judgments based on Profile condition. The data did not reveal any statistically significant effects of Profile on any of the measured variables, *F*s < 1, *p*s > .44 (for additional analyses, see **S6 Text**).

To provide additional support for the findings of no differences in user judgments based on specific elements, the frequentist analyses were complemented with a Bayesian approach. Testing the assumption of no difference from the null hypothesis, the Bayesian ANOVA conducted on rent decisions between the three Profile conditions revealed a Bayes factor of BF_01_ = 9.05, indicating that the data was around 9 times more likely under the null than the alternative hypothesis. For confidence, a BF_01_ = 13.35 was found in favor of the null. With regards to host ratings, for sociability a BF_01_ = 14.36 was found, for trustworthiness a BF_01_ = 14.01 was found, and for credibility a BF_01_ = 12.15 was found.

### Discussion

The data consistently shows that the information contained in the individual TRI elements does not influence judgment beyond their simple presence. No differences in user judgements were found when comparing triplets that users selected most often (3-Wanted), or the ones they avoided selecting (3-Avoided), or simply showing three random elements (3-Random). The findings suggest that providing users with at least three TRI elements to aid their decision-making is sufficient to produce a strong positivity in their judgements of hosts on SE accommodation-style platforms.

## General discussion

The SE’s proliferation brings opportunities for individuals, but also potential, and yet unknown, risks. The information individuals rely on in SE platforms differs significantly from what consumers generally rely on in traditional marketplaces. Namely, there is an overreliance on UGC to assess the trustworthiness and reputation of other users. In this respect, the novelty of the SE and its multiple platforms raises many questions regarding people’s judgments and their ability to use such information to make informed decisions.

To understand the impact of trust and reputation based systems on human judgment, the current paper investigated the role of DI information on user judgments in an artificial SE accommodation platform. Over three studies, the data showed that users are strongly influenced by the presence of TRI, resulting in an overall positivity towards hosts and increased tendency to rent rooms. The ratings users gave to hosts on measures of sociability, credibility, and trustworthiness increased significantly when they were provided information relating to the hosts’ DI, compared to seeing a profile lacking such information. However, the amount or specificity of this information did not seem to impact judgement.

The findings of Study 1 speak to the effects that P2P platforms have on people’s decision-making process. In line with past research, TRI is found to have a significant impact on users’ decision to engage with others on P2P platforms, and to seek their services [30]. Here, we find that even when maintaining the quality of the service or product constant (i.e. the rooms), users perceive these in a more positive light if they are provided with TRI regarding the provider (i.e. host).

The sharp contrast in rent decisions between the Hidden and other profile conditions would suggest that TRI is used in deciding not only our perceptions of other peers on SE platforms, but also of the likelihood we will use their goods or services. Viewing the Hidden condition profiles as reflecting new users on a platform, illustrates that even profiles devoid of TRI can have success (as seen by the overall high rent decisions). However, it is clear that profiles containing platform- and community-generated information have a significant advantage [31,37,40]. Interestingly, though, we found cross-platform reputation not to be a frequently selected item in the Reveal condition, which suggests that users retain a preference for information generated “locally” by the community of the platform of interest. Furthermore, the lack of a statistically significant difference between the Visible and Reveal conditions implied either that users were discounting the extra information, resulting in no added positivity beyond that seen in the Reveal condition, or that the act of selecting which information to reveal compensated for the information difference.

Study 2 examined these explanations. Here, the data suggested that simply seeing three elements is sufficient to generate an overall positivity towards hosts and to increase rental decisions, while the act of self-selecting information has no effect on judgment.

Study 3 investigated whether specific information mattered towards this positivity; that is, whether different elements result in a different perceptions of hosts. Overall, the data strongly showed that specific combinations did not matter. Irrespective of the frequency with which users tend to select them naturally, presenting three TRI elements is sufficient to positively impact judgments and increase rent decisions.

The above findings resonate with the judgment and decision-making literature, expanding it to online user behavior. From this literature, we know that people are cognitive misers [55], rarely able to incorporate diverse information from multiple sources into their judgements. People are also subject to several biases that can influence their perception and the trust they place in others, as well as the risks they are willing to take. Rather than relying on rational and strategic process to make decisions, people tend to rely on quick and automatic rules-of-thumb to make their judgments [42]. Moreover, people are poor at estimating their own preferences for information [43], and are limited to around three cues when making judgments [44].

Thus, while people show a strong selection preference for specific TRI elements in SE environments, these may not reflect specific differences in how the information is perceived or used by individuals. Indeed, even in novel environments, where people have minimal information on how different information affects outcome, they seem to develop strong preferences over time (see coherent arbitrariness [56]). They may, nonetheless, prefer TRI due to social convention (i.e. they use it because others seem to use it [57] or because they are accustomed to relying on it [58]).

Lastly, the literature argues that TRI serves to reduce the uncertainty experienced in SE environments [3,40]. However, across all studies users’ decision confidence was found to be stable and overall high, regardless of the amount or type of TRI presented. The data favors our interpretation that TRI produces a “positivity effect” on user judgement, rather than an “uncertainty-reduction effect” (cf. [59]).

As a possible limitation to our findings and their implications (see next Section), it is worthwhile to remember that our research was an initial exploration into user judgment in the SE. In order to minimize spurious effects from extraneous variables a number of controls, detailed in the Methods Sections, were implemented in constructing our SE platform, while still maintaining ecological validity with respect to its real-world counterparts. Yet, it can be argued that our study used incentives that were hypothetical (i.e., lower than those in real-life) rather than salient (i.e., comparable to those in real life). Is this likely to have mattered?

Within the literature on human judgment (across multiple fields; psychology, decision-making, economics, etc.) the role of stakes (or motivation/incentive/rewards) has been found to be rather complex. For instance, Camerer and Hogarth [60] reviewed 74 studies where incentives were manipulated. The modal finding of their analysis was that incentives had no effect on mean performance but affected performance variance instead. In 15 of the studies, there was no performance standard but, in eight of them, higher incentives made participants more risk-averse. Holt and Laury [61] similarly showed how the same task can result in different decision-making behavior based on the stakes surrounding the task. In cases with performance standards, Camerer and Hogarth found very mixed evidence: 27 studies showed no effect of incentives, 23 showed they facilitated performance, and nine showed they impaired performance. Incentives helped when better performance could be achieved by applying greater effort: such tasks include those that involve memory recall (e.g., Kahneman & Peavler, [62]), binary choice, easy problem solving, and simple clerical tasks. Conversely, incentives can have a detrimental effect on complex tasks (as demonstrated by research on “choking under pressure”). Financial incentives also have no effect when a task is very easy to perform well (ceiling effects), when it is very hard to improve performance (floor effects), and when the intrinsic rewards from participating in the task are already high. Many sequential bargaining and game-theoretical tasks come into these categories.

This begs the question: which category does our study fall within? If we consider the study to be without a performance standard, the research mentioned above suggests that people would be more risk-averse in their choices if they were more consequential (as in a real-life scenario). However, we have no way of determining whether our participants were making risk-seeking choices in the first place. If we consider a higher selection of properties offered by more trustworthy hosts as an indication of higher performance, it is unlikely on the basis of Camerer and Hogarth's [60] conclusions that higher incentives would have changed this. Our task was complex, but it did not involve memory, and while participating in it provided some intrinsic rewards, participants were not rewarded/penalized based on outcome.

### Implications

Decision-making in an online P2P environment can be a complex task. This process is compounded by issues with the information provided to users to aid in their decision-making that exist in the SE, two central ones being the overall positivity of such information and, consequently, its low diagnosticity [4,25]. Indeed, ratings on SE platforms show a stronger bias towards high ratings than on other P2P platforms [4,47], which severely reduces their usefulness to users.

Despite this, there is a trend for increased UGC on online platforms. Users seem more willing to provide such information, even when private and potentially identifying, and platforms themselves are incentivizing this type of information-sharing [63]. But, our data show that more of such information may not assist people in any meaningful way, which in turn suggests that both platforms and their users gain no benefit from collecting and sharing more information than is currently available. However, these considerations do not necessarily imply that limiting the proliferation of UGC would have no consequence.

First, research finds that users trust UGC more than objective metrics when making their decisions, and carry more weight in the decision-making process than other forms of information [30,64]. This is supported by the current data, finding that users show a preference for selecting elements that result from the aggregate ratings or other users’ testimonials (e.g., guest reviews), compared to platform-generated information (e.g., host verification).

Second, attempting to reduce the amount of TRI from existing or emerging platforms may backfire in terms of user perception. Users may *expect* specific information to be present (even if not used), with its absence leading to more negative appraisals or to avoidance [65].

Past research has argued that reputation systems and user-generated reviews may have the primary purpose of allowing users to learn more about each other before engaging in any interactions or transactions, acting as a monitoring and policing system [5,66,67]. However, the current findings demonstrate that this information can also act as a strong influencer in the perceptions of others, leading users to see peers in a more positive light.

A consideration that emerges from the current findings relates to the concept of online privacy and how people construct their digital identity. A culture of information-sharing is forming, under the guise of more informed decision-making, which may force individuals wanting to participate in these communities to share private and sensitive information without any benefit to the community or individual decision-making outcomes. The current data show it is unnecessary for users, beyond a certain point, to provide such information, and may even prove detrimental in the long run. In this respect, we advise caution in how SE platforms choose to expand and implement their reputation-based systems.

### Future directions

As it has been argued [47], the current “5-for-5” culture in ratings drastically reduces the overall diagnosticity of TRI in favor of social cohesion and community participation. This motivated our choice to limit the variance in the TRI shown to users to reflect real-world data (see **S2 Text**). Within this specific context our findings show that the *presence* of some TRI, rather than certain specific elements, is the main driver in trust between SE users. Future investigations should attempt to ground these findings in established theoretical models to provide a wider context and understanding of user psychology. For instance, social exchange theory [68] and reward motivation theory [69,70] may be useful to shed light on the currently observed—paradoxical—behavior, according to which SE users overall decrease the information content and diagnosticity of TRI on platforms, while uncritically relying on its mere presence to make decisions.

The aim of the current research was not to assess whether specific reputation and trust elements are useful to discriminate among different options, but rather to understand how the *presence* of such information affects user judgement and decision-making in a setting that closely follows real-world patterns.

A natural extension of the current research is to understand how accurate individuals are at classifying profiles based on their quality. The current design considered the effect of cue diagnosticity to the extent that no one particular element provided specific information to classify a room as “good” or “bad”, but aimed to reflect the natural distribution seen on SE platforms [4,47]. However, if the profiles users saw varied more in terms of quality and uncertainty, would ratings reflect these differences or would they continue to show a positivity effect? Introducing variability and an element of diagnosticity into the information users see on such profiles may provide a more complete image of human behavior in the SE, for example varying the type of profiles users see or analyzing behavioral patterns of “low-raters” (e.g., hyper-critical or ‘picky’ users) and “high-raters” (e.g., uncritical or lenient users). Similarly, this can extend into considering the difference in effect and strength that negative information has on judgments compared to positive information [71]. This research is currently being undertaken.

Due to explorative nature of our studies, we did not attempt to synthesize our results into a trust model along the lines, e.g., of the work by Meyer et al. [20] or more recent developments in the Internet setting (see, e.g., [72]). Yet, we believe that our work, and the aforementioned ongoing research, represent an important step towards the development of a trust model for interactions in the SE.

Lastly, our work does not take into account the role of platforms as mediators of trust. Indeed, it has been shown [12] that trust towards a specific platform correlates positively with trust between its users. While we deliberately eliminated any explicit reference to real-world SE platforms (e.g., Airbnb) from our artificial accommodation platform in order to suppress any possible spurious enhancement in the perceived trustworthiness of hosts, it would be very interesting to replicate our studies in specific platforms as a way to indirectly measure their additional impact on trust.

### Conclusion

Currently, the effect of TRI on user behavior in the SE was investigated. The focus was on how presenting users with information about hosts’ DI, in an accommodation SE platform, would impact their perceptions of hosts and the likelihood of renting their private rooms. Over three studies, the data consistently shows that users find hosts whose profiles display TRI as more trustworthy, credible, and sociable. More importantly, they also rent more properties if such information exists. Despite users showing a consistent and strong preference for specific information, they demonstrate this positivity in judgement from seeing any three elements relating to the hosts’ DI. These findings illustrate how TRI can affect user decision-making, cautioning people on the risks of relying too heavily on this information. Research should carefully consider how information relating to trust and reputation on SE platforms can impact user judgement.

## Supporting information

S1 Text Additional measures. (PDF)

S2 Text Profile elements. (PDF)

S3 Text Study 1 Demographics and supplementary analyses. (PDF)

S4 Text Triplet analysis. (PDF)

S5 Text Study 2 Demographics and supplementary analyses. (PDF)

S6 Text Study 3 Demographics and supplementary analyses. (PDF)

S7 Text Data processing ReadMe. Explanation of the data processing used for the three reported studies and description of variables in the datasets.
(PDF)
