# Supplementary material for: Digital Identity: The effect of trust and reputation information on user judgement in the Sharing Economy
Source: PLoS One. 2018 Dec 13;13(12):e0209071. doi: 10.1371/journal.pone.0209071 (PMC6292641; doi:10.1371/journal.pone.0209071)
Supplement: S1 Text — (PDF) [file pone.0209071.s001.pdf]

S1 Additional Measures

Pre-task questions:

Number of memberships on Sharing Economy sites/apps (e.g. Uber, Airbnb, TaskRabbit)

- ◯ None
- ◯ 1 - 2
- ◯ 3 - 4
- ◯ 5 - 10
- ◯ >10

Thinking about your usage and experience with sharing economy platforms (e.g., Airbnb, Uber), please answer the below questions regarding your behaviour and history on such sites/apps:

How long have you been a member of or using such sites/apps?

<1 month	2 - 6 months	8 - 10 months	12 months	>3 years

How many times have you used such sites/apps within the last year?

0 - 5	5 - 10	10 - 20	20 - 30	>30

In general, how satisfied have you been with using such platforms?

**Not at all Satisfied**	1	2	3	4	5	6	7	8	9	10	**Very Satisfied**

How would you rate your sense of belonging to such peer-to-peer platforms?

**None at all**	1	2	3	4	5	6	7	8	9	10	**Very Strong**

Please read the below statments, and rate how strongly you agree or disagree with them:

1 - "Being accepted as a member of a group is more important than having autonomy and independence."

**Strongly Disagree**	1	2	3	4	5	6	7	8	9	10	**Strongly Agree**

2 - "Being accepted as a member of a group is more important than being independent."

**Strongly Disagree**	1	2	3	4	5	6	7	8	9	10	**Strongly Agree**

3 - "Group success is more important than individual success."

**Strongly Disagree**	1	2	3	4	5	6	7	8	9	10	**Strongly Agree**

4 - "Being loyal to a group is more important than individual gain."

**Strongly Disagree**	1	2	3	4	5	6	7	8	9	10	**Strongly Agree**

5 - "Individual rewards are not as important as group welfare."

**Strongly Disagree**	1	2	3	4	5	6	7	8	9	10	**Strongly Agree**

6 - "It is more important for a manager to encourage loyalty and a sense of duty in subordinates than it is to encourage individual initiative."

**Strongly Disagree**	1	2	3	4	5	6	7	8	9	10	**Strongly Agree**

Post-task questions:

Please answer to the best of your abilities.

When usually making rental decision online, which information source carries the most weight in your decision to "Rent" or "Not rent"? **(select 3 max)**

- ☐ Picture of the room
- ☐ Host's profile image
- ☐ Description of the room
- ☐ Profile's Star Rating
- ☐ Total number of Reviews
- ☐ Online Reputation Scores
- ☐ Social Media Presence information
- ☐ Reviews from other Hosts
- ☐ Reviews from other Guests
- ☐ My Intuition
- ☐ None of the above

If you ticked "None of the above", please write down how you made your judgements:

To help in making such rental decisions, would you like to see more cross-platform information about the hosts? *(i.e. their reputation on other peer-to-peer platforms, or their social media activity information)*

- ○ Yes
- ○ No
- ○ Not sure

Please write down any other information you felt was missing from the profiles which may have helped in your decision making: *[optional]*

What do you think the aim of this experiment was?

Additional Dependent Variables:

Cognitive trust and Affective trust towards the hosts were also measured during the three studies. However, preliminary analyses indicated strong overlap with the overall Trustworthiness measure. The addition of this variable in analyses or the results provides no further insight into user judgment in this specific paradigm, so were not presented in the main text. The questions used to assess these measures are as follows:

- **Cognitive Trust**: ‘Given the information displayed on the webpage, I have no doubt about this host’s competence’’ (10-point Likert Scale)
- **Affective Trust**: ‘If I shared my concerns about the property with this host, I know s/he would respond constructively’’ (10-point Likert Scale)
