# Supplementary material for: Digital Identity: The effect of trust and reputation information on user judgement in the Sharing Economy
Source: PLoS One. 2018 Dec 13;13(12):e0209071. doi: 10.1371/journal.pone.0209071 (PMC6292641; doi:10.1371/journal.pone.0209071)
Supplement: S2 Text — (PDF) [file pone.0209071.s002.pdf]

S2 Profile Elements

Below is a description of each element comprising the profiles of hosts shown to participants, along with an example of how it appeared in the experiments.

Room Photo

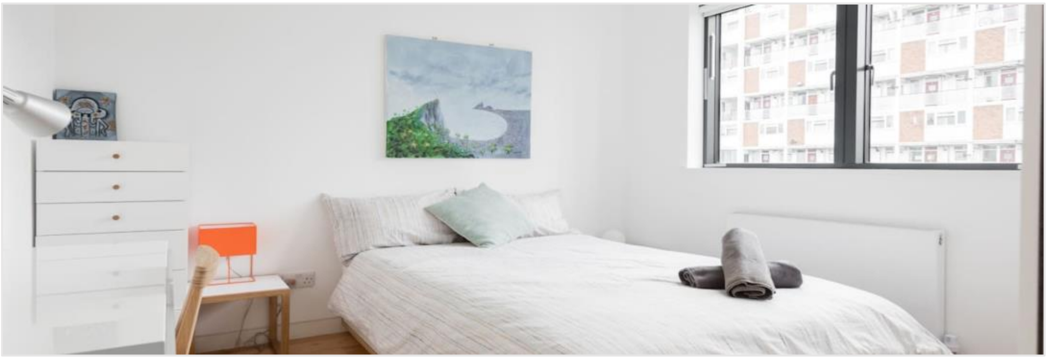

Creation: The room images were selected from Airbnb. These were limited to a single borough of London, within one standard deviation (SD) of the average price for a room in the area, equating quality and amenities offered.

Title

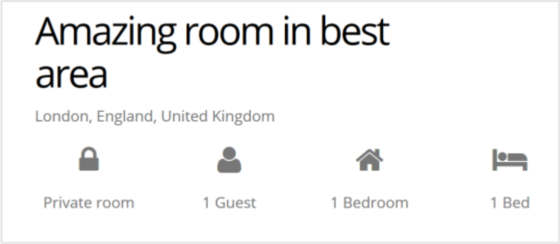

Creation: Selected directly from Airbnb. These were controlled for word length (3-7), location information, and other identifying cues.

Description of room

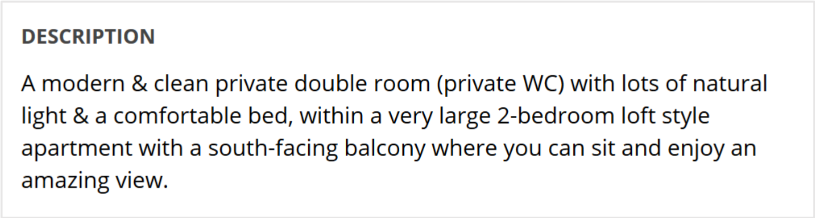
DESCRIPTION

A modern & clean private double room (private WC) with lots of natural light & a comfortable bed, within a very large 2-bedroom loft style apartment with a south-facing balcony where you can sit and enjoy an amazing view.

Creation: Room descriptions were taken from Airbnb. These focused on descriptive information, did not contain identifying location cues or opinions, and were constrained to between 20-50 words.

Host Photo

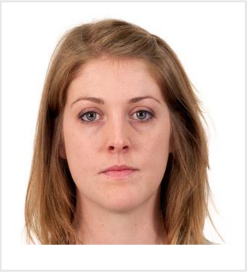

Creation: The profile photos were created from the Chicago Face Database, which offers descriptions of each photo with ratings on several factors [1]. Here, facial dominance, trustworthiness, attractiveness, and ethnicity were controlled, as differences in facial traits can influence decision-making (e.g., [2,3]. Only images that did not deviate more than ±1 SD from the mean on each characteristic, within one ethnicity (White-Caucasian), were selected. The same care was given to the "guest" and "host" reviews photos, without limiting ethnicity.

Star Rating

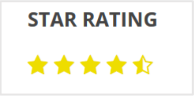

Creation: The star ratings on the profiles were dynamically and randomly generated for each profile. The stars ranged from 1 to 5 in half-star increments, however, the distribution was limited to 4 to 5 stars, as is typical of such profiles [4,5].

Host Verification

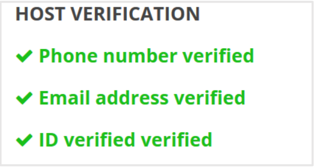

Creation: These represented the platform's verification of the host's identity. This always

showed a green tick-mark for all three verifications available: mobile, ID, and address.

Number of Reviews

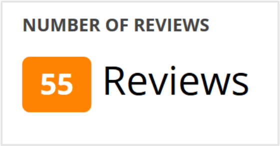

Creation: The number of reviews was displayed as a badge with a number inside. This represented the total number of reviews that existed for the specific room, acting as a metric for reputation and longevity. The number was randomly generated starting with 50 + a random number from 0-30.

Guest Reviews

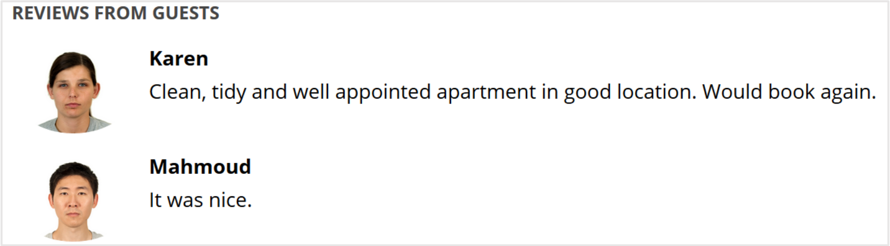

Creation: The guest and host reviews were taken directly from Airbnb. Guest reviews reflect the testimony of past users that have stayed with that particular host, while host reviews are testimonials from other hosts on the platform that have also stayed with that host in the past. The comments focused on the room quality, contained no identifying information, and did not exceed 15 words.

Host Reviews

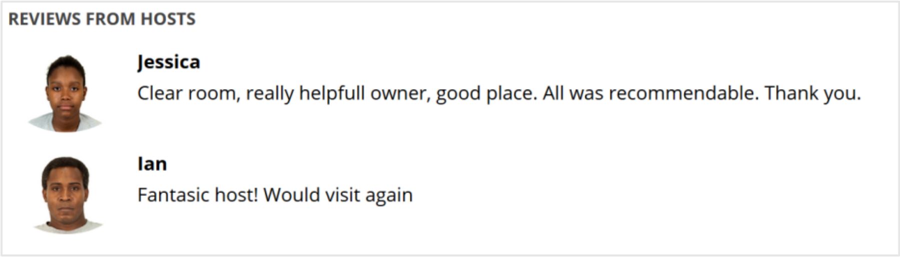

Creation: See Guest Reviews.

Social Media Presence

SOCIAL MEDIA PRESENCE

Twitter	Facebook	Google+
2k+ Mentions	700+ Friends	200+ +1s
20+ Lists	0.5k+ Likes	200+ Comments
1.5k+ Retweets	700+ Comments	200+ Reshares
2k+ Followers	200+ Wall Posts	

Info is gathered from public posts only

Creation: Social media presence was composed of the host's online presence on Twitter, Facebook, and Google+, generated randomly for each profile. For Twitter, the data included the number of "mentions" for the host (range 1 - 2k, with 1 decimal place (dp) increments), number of "lists" (range 10-30), number of "retweets" (range 1 – 2k, 1 dp), and number of "followers" (range 1 – 2k, 1dp). For Facebook, the data included number of "friends" (range 300 – 700), "likes" (range 0.5 – 1.5k, in 1 dp increments), "comments" (range 400 – 700), and "wall posts" (range 100 – 200). For Google+, the data was comprised of number of "+1's" (range 200 – 400), "comments" (range 100 – 200), and "reshares" (range 100 – 200).

Online Market Reputation

ONLINE MARKET REPUTATION

ebay	User rating: 5/5★<br>Positive Feedback: 97%
UBER	Rider Rating: 4/5

Creation: Online market reputation containing the host's user profile ratings on two other platforms, eBay and Uber. For eBay this represented the "user rating", as a randomly-generated fractional rating from 1 to 5 (effective range 4 – 5, with .5 increments; e.g., 4/5), and "positive feedback" with a percentage randomly generated (90 + random number between 0 – 10). For Uber the information contained the "rider rating", with a number ranging from 1 to 5, but a constrained range of 4 – 5 (.5 increments).

Instructions (example from the Reveal Condition):

Welcome!

The study consists of 10 trials. For each trial you will be shown a Host's profile for a Private Room they wish to rent out.

For each profile, you need to decide whether you want to "Rent" or "Not Rent" the room, and state your opinion on a few items.

First, you will see an Example profile. This is meant to familiarise you with the look of the trials and the information available.

Please look carefully at the entire profile, and answer all the questions presented.

There is no time limit for the decision.

After each profile there will be a validation question, making sure you were attentive to the information displayed.

In each Trial many of the elements on the Profiles are hidden. You will need to spend a COIN to reveal them by clicking on them. You have 3 COINS to spend on each profile.

You will have to decide how to spend these coins to reveal the information you consider is important for making your decision.

For the Example you will have unlimited coins to spend, to familiarise yourself with the type of information available.

We will start with a practice trial

Start

Profile Questions:

Do you want to rent this property?

Yes	No

How confident are you in your decision to Rent/Not rent?

Not at all Confident	1	2	3	4	5	6	7	8	9	10	Very Confident

How sociable do you think the host would be?

Not at all Sociable	1	2	3	4	5	6	7	8	9	10	Very Sociable

How would you rate the trustworthiness of the host?

Very Untrustworthy	1	2	3	4	5	6	7	8	9	10	Very Trustworthy

How credible do you think the information about this room is?

Not at all Credible	1	2	3	4	5	6	7	8	9	10	Very Credible

Given the information displayed, how would you rate the competence of the host?

Not at all Competent	1	2	3	4	5	6	7	8	9	10	Very Competent

If you shared your concerns about the property with this host, do you think s/he would respond constructively?

Not at all Constructively	1	2	3	4	5	6	7	8	9	10	Very Constructively

Submit

References

1. Ma DS, Correll J, Wittenbrink B. The Chicago face database: A free stimulus set of faces and norming data. Behavior research methods. 2015;47: 1122–1135.

2. Edelman BG, Luca M. Digital discrimination: The case of Airbnb. com. Harvard Business School NOM Unit. 2014;Working Paper no: 14–054.

3. Edelman BG, Luca M, Svirsky D. Racial Discrimination in the Sharing Economy: Evidence from a Field Experiment. American Economic Journal: Applied Economics. 2017;9: 1–22. doi:10.1257/app.20160213

4. Livan G, Caccioli F, Aste T. Excess reciprocity distorts reputation in online social networks. Scientific Reports. 2017;7. doi:10.1038/s41598-017-03481-7

5. Zervas G, Proserpio D, Byers J. A first look at online reputation on Airbnb, where every stay is above average. 2015;
