# Supplementary material for: Digital Identity: The effect of trust and reputation information on user judgement in the Sharing Economy
Source: PLoS One. 2018 Dec 13;13(12):e0209071. doi: 10.1371/journal.pone.0209071 (PMC6292641; doi:10.1371/journal.pone.0209071)
Supplement: S3 Text — (PDF) [file pone.0209071.s003.pdf]

S3 Study 1 Demographics and Supplementary Analyses

Participant Demographics

Ethnicity						
**Asian**	**Black**	**Latino**	**White**	**Multi-racial**	**Other**	**N**
6	14	5	98	0	0	123

Sharing Economy - Memberships					
**None**	**1-2**	**3-4**	**5-10**	**>10**	**N**
19	82	19	2	1	123

Sharing Economy – Usage Length					
**<1 month**	**2-6 months**	**8-10 months**	**12 months**	**>3 years**	**N**
29	22	19	35	18	123

Sharing Economy - Usage Frequency					
**0-5**	**5-10**	**10-20**	**20-30**	**>30**	**N**
67	26	17	8	5	123

Sharing Economy - Satisfaction									
**1**	**3**	**4**	**5**	**6**	**7**	**8**	**9**	**10**	**N**
6	2	4	23	8	19	38	14	9	123

Sharing Economy – Sense of Belonging									
**1**	**3**	**4**	**5**	**6**	**7**	**8**	**9**	**10**	**N**
16	6	13	5	28	16	13	12	8	123

Gender Manipulation Check

An initial analysis considering gender differences based on the Profile condition was conducted, however, this did not reveal any significant effects on any of the measured DVs, *p*s > .05.

Bayesian Analysis

To add more support for the explanation that there are no differences between the Visible and Reveal conditions, the frequentist analysis was supplemented with a Bayesian analysis. Multiple independent-samples t-tests were conducted under the assumption of no difference between the two conditions. Below is a table detailing the exact values for each dependent variable measured.

Bayesian Independent Samples T-Test

	BF_{01}	error %
Rent	2.611	0.022
Confidence	4.340	0.024
Social	4.327	0.024
Trust	4.219	0.024
Credible	4.316	0.024
