# Supplementary material for: Digital Identity: The effect of trust and reputation information on user judgement in the Sharing Economy
Source: PLoS One. 2018 Dec 13;13(12):e0209071. doi: 10.1371/journal.pone.0209071 (PMC6292641; doi:10.1371/journal.pone.0209071)
Supplement: S4 Text — (PDF) [file pone.0209071.s004.pdf]

S4 Triplet analysis

We assess the statistical significance of the three elements selected by users in the Reveal condition by contrasting them with those that would have been observed under a null hypothesis of random selection (i.e. a pattern of random “token spending”).

In order to assess the statistical significance of these findings, we assumed a null hypothesis of random selection, i.e. assuming that each trial results from the random selection of 3 out of the 7 available elements. This allows to assign a p-value to each triplet. Assuming a certain triplet has been selected x times, this reads,

$$P_u(x) = 1 - \sum_{i=0}^{x-1} \binom{n}{i} p^i \, (1-p)^{n-i} \, ,$$

where n denotes the total number of trials (42 participants x 10 trials each = 420), and p denotes the probability of selecting any particular triplet at random out of the 7 available options, which reads

$$p = {}^{1}\!/\!\binom{7}{3} \sim 0.0286.$$

The above equation for P(x) denotes the complementary cumulative function of the binomial distribution, and computes the probability of observing the triplet under consideration x *or more* times. Symmetrically, the above framework can be used to assess the possible under-representation of certain triplets in the choices made by users. Namely, the p-value

$$P_d(x) = \sum_{i=0}^{x} \binom{n}{i} \, p^i \, (1-p)^{n-i}$$

evaluates the probability of observing a certain triplet x or less times under a null hypothesis of random selection.

We test significance at a univariate significance level of 1%, and we perform a Bonferroni correction to the above p-values to take into account the multivariate nature of our test (420 trials). When testing for the over-representation of triplets, we find 3 combinations

to be statistically significant at the 1% univariate level. These are: “stars + guest reviews + number of reviews”, “stars + guest reviews + host verification”, or “stars + guest reviews + host reviews”.

When instead testing for the under-representation of triplets, we find 5 combinations to be significant at the 1% univariate significance level, which correspond to the only combinations never selected in any trial. Three of these were found to feature both the “Host Verification” and “Host Reviews” items, with the third one being, respectively, “Social Media Presence”, “Online Market Reputation”, or “Number of Reviews”. This motivated our selection of triplets for Study 2.

We also employ a similar methodology to assess whether participants progressively converge over a set of preferred choices over time. In order to do this, we compare the overlap between choices in consecutive trials and, again, compare it to a null hypothesis of random selection. In particular, for each trial we count the number of participants whose triplet shares at least two items with the triplet of the previous trial. In analogy with the above p-values, we compute the probability of observing c or more users whose triplet shares at least two items with their previous choice as

$$P_t(c) = 1 - \sum_{i=0}^{c-1} \binom{u}{i} \pi^i (1-\pi)^{c-i},$$

where u = 42 is the number of users and

$$\pi = \frac{1+\binom{3}{2}\binom{4}{1}}{\binom{7}{3}} \sim 0.371$$

is the probability of selecting at least two of the 3 items selected in the previous triplet at random. In the plot below we show such p-values for each set of trials, and we also report the corresponding Bonferroni corrected significance level. As it can be seen, we observe a statistically significant overlap between consecutive trials already from trial 2, illustrating a strong tendency to select the same items from the very beginning with very little exploration of other possibilities.

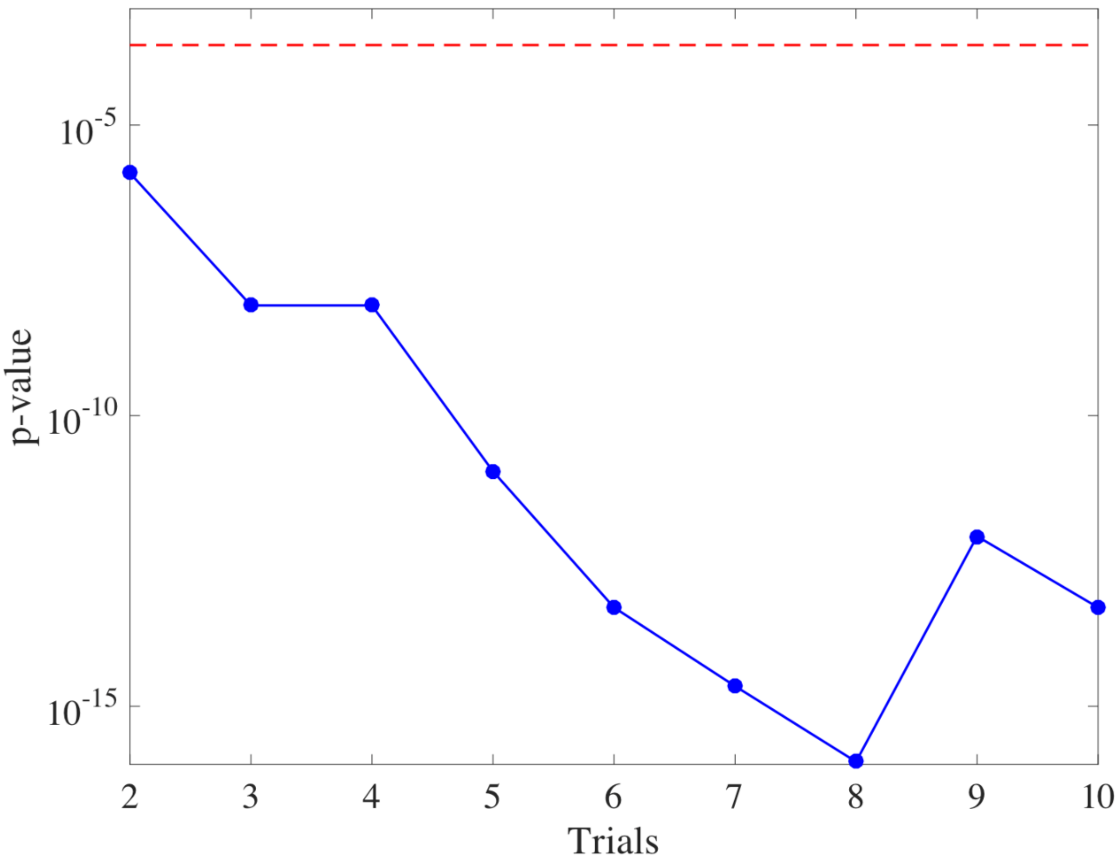

Fig 1. The plot illustrates the statistical significance of overlap in element selection between consecutive trials (i.e. if the selection of elements in one trial predicted the selection in the next trial). The x-axis denotes the overlap in selection between trial X and trial X-1. The y-axis represents the p-value of the overlap. The blue solid line shows the p-values for each trial comparison, while the red dashed line represents the Bonferroni corrected p-value level of significance.
