# Supplementary material for: Digital Identity: The effect of trust and reputation information on user judgement in the Sharing Economy
Source: PLoS One. 2018 Dec 13;13(12):e0209071. doi: 10.1371/journal.pone.0209071 (PMC6292641; doi:10.1371/journal.pone.0209071)
Supplement: S5 Text — (PDF) [file pone.0209071.s005.pdf]

S5 Study 2 Demographics and Supplementary Analyses

Participant Demographics

Ethnicity						
**Asian**	**Black**	**Latino**	**White**	**Multi-racial**	**Other**	**N**
9	11	1	90	6	9	117

Sharing Economy - Memberships					
**None**	**1-2**	**3-4**	**5-10**	**>10**	**N**
10	82	20	5	0	117

Sharing Economy – Usage Length					
**<1 month**	**2-6 months**	**8-10 months**	**12 months**	**>3 years**	**N**
12	17	19	48	21	117

Sharing Economy - Usage Frequency					
**0-5**	**5-10**	**10-20**	**20-30**	**>30**	**N**
53	30	18	6	10	117

Sharing Economy - Satisfaction									
**1**	**3**	**4**	**5**	**6**	**7**	**8**	**9**	**10**	**N**
5	3	1	11	10	23	30	21	13	117

Sharing Economy – Sense of Belonging									
**1**	**3**	**4**	**5**	**6**	**7**	**8**	**9**	**10**	**N**
10	4	6	5	15	17	23	23	9	117

Manipulation Check

Preliminary analyses revealed no statistically significant differences between the three sub-conditions of the 3-Seen condition ("stars + guest reviews + number of reviews", $n = 22$, "stars + guest reviews + host verification", $n = 20$, and "stars + guest reviews + host reviews", $n = 19$), *p*s > .05; thus, all subsequent analyses collapsed across the sub-conditions.

Subsequently, an analysis considering gender differences based on the Profile conditions was conducted. This did not reveal any significant effects on any of the measured DVs, *p*s > .05.

Finally, to verify that the responses from Study 2's 3-Reveal condition ($n = 54$) did not differ from those of Study 1's Reveal condition ($n = 42$), the data of the two was compared on all measured DVs. In all cases, no significant differences were found between the two conditions, *t*s < 1, *p*s > .05.

Bayesian Analysis

Independent-samples t-tests were conducted investigating the non-significant findings between the 3-Seen and 3-Reveal conditions uncovered when using the frequentist approach. These analyses were conducted on all measured DVs: rent decision, confidence in decision, trustworthiness, credibility, and sociability. As suggested, the data suggests that user behaviour did not differ if either allowed to select three TRI elements to aid their decision or simply be provided three elements.

Bayesian Independent Samples T-Test		
	BF_{01}	error %
Rent	2.288	0.017
Confidence	3.029	0.012
Sociability	3.768	0.007
Trust	2.034	0.018
Credibility	2.855	0.013

Comparison with Study 1

To fully understand the effect of seeing only three elements when making a decision, it is relevant to compare with the data in Study 1's Hidden and Visible condition. This allows for a clear comparison to be made of the effect of amount of information on participants' decision-making.

Comparing the data from the 3-Seen and 3-Reveal with the Hidden condition revealed significant differences in participants' responses. A main effect of Profile condition was seen for the rent decision, $F(2, 154) = 4.27$, $p = .016$, $\eta^2 = .053$. Post-hoc Tukey HSD comparisons revealed a significant difference between the Hidden and the 3-Reveal condition ($M = 7.93$, $SD = 2.18$), $p = .011$, echoing Study 1. However, there was no differences in scores between the 3-Seen ($M = 7.34$, $SD = 2.58$) and 3-Reveal condition, or 3-Seen and Hidden condition. With respect to confidence ratings, no significant difference was found based on profile condition, F < 1, ns.

Considering credibility, differences were found based on profile condition, $F(2, 154) = 4.30$, $p = .015$, $\eta^2 = .053$. Post-hoc comparisons revealed a significant difference between the Hidden and 3-Reveal condition ($M = 75.25$, $SD = 13.18$), $p = .012$, and a marginally significant difference between the Hidden and 3-Seen condition ($M = 73.00$, $SD = 11.80$), $p = .092$.

For trust towards the host, a main effect of profile was found, $F(2, 154) = 7.09$, $p = .001$, $\eta^2 = .084$. Post-hoc comparisons revealed a significant difference between the Hidden

condition and both the 3-Seen (*M* = 70.70, *SD* = 11.37), *p* = .023, and the 3-Reveal (*M* = 73.77, *SD* = 13.79), *p* = .001.

A marginally significant effect of profile was also seen for sociability, $F(2, 154) = 2.73$, $p = .069$, $\eta^2 = .034$. Post-hoc comparisons revealed only a marginally significant difference between the Hidden condition and the 3-Reveal condition (*M* = 67.68, *SD* = 15.45), *p* = .060.

Finally, an analysis comparing Study 2's conditions with Study 1's Visible condition was performed, assessing differences in user judgement when more information was available. This did not reveal any significant differences on any of the measured DVs, *p*s > .13.

Thus, when comparing with Study 1's Hidden condition it was found that the same positivity towards hosts was observers in users' data. But, when comparing to Study 1's Visible condition, no differences were found. This supports the prediction that users focus/use primarily on around 3 elements when making rental choices and judging hosts.
