# Supplementary material for: Digital Identity: The effect of trust and reputation information on user judgement in the Sharing Economy
Source: PLoS One. 2018 Dec 13;13(12):e0209071. doi: 10.1371/journal.pone.0209071 (PMC6292641; doi:10.1371/journal.pone.0209071)
Supplement: S6 Text — (PDF) [file pone.0209071.s006.pdf]

S6 Study 3 Demographics and Supplementary Analyses

Participant Demographics

Ethnicity						
**Asian**	**Black**	**Latino**	**White**	**Multi-racial**	**Other**	**N**
18	16	12	139	0	4	189

Sharing Economy - Memberships					
**None**	**1-2**	**3-4**	**5-10**	**>10**	**N**
24	129	34	2	0	189

Sharing Economy – Usage Length					
**<1 month**	**2-6 months**	**8-10 months**	**12 months**	**>3 years**	**N**
32	38	27	52	40	189

Sharing Economy - Usage Frequency					
**0-5**	**5-10**	**10-20**	**20-30**	**>30**	**N**
85	55	26	6	17	189

Sharing Economy - Satisfaction									
**1**	**3**	**4**	**5**	**6**	**7**	**8**	**9**	**10**	**N**
10	1	6	25	17	38	44	29	19	189

Sharing Economy – Sense of Belonging									
**1**	**3**	**4**	**5**	**6**	**7**	**8**	**9**	**10**	**N**
20	8	9	14	25	20	36	32	16	189

Manipulation Check

As in Study 2, preliminary analyses found no differences between any of the sub-conditions used in the study, either Avoided ("social media presence", n = 22, "online market reputation", n = 25, or "number of reviews", n = 20) or Wanted ("host reviews", n = 24, "host verification", n = 24, "number of reviews", n = 29), for any of the measured DVs, Fs < 1, ps > .05.

Several independent-samples t-test were conducted to ensure that the 3-Wanted condition was not significantly different from the Study 2's 3-Seen condition. Considering each DV, no significant differences were found, ts ≤ 1, ps > .05, suggesting the conditions can be treated equally.

Finally, a gender analysis between the three conditions did not reveal any statistically significant differences on any of the measured DVs, Fs ≤ 1, ps > .05.

Comparison with Study 1

To understand if the triplets seen by participants in the three profile conditions had an effect on their ratings of hosts and decisions to rent, the data from Study 3' 3-Random and 3-Avoided was compared with the Hidden, Reveal, and Visible conditions from Study 1. This allows for a direct comparison of how the type and amount of TRI impacts users' decision-making.

Random vs Hidden. Conducting independent-samples t-tests between the 3-Random and S1-Hidden condition for each relevant DV revealed significant differences in line with

our predictions.

For rent decisions, a significant difference was found where users in the 3-Random (*M* = 8.16, *SD* = 2.34) condition rented on average more private rooms than those in the S1-Hidden condition, $t(94) = 3.43$, $p = .001$, 95% CI [.71, 2.66], $d = 0.71$.

For confidence, no difference was found between the two conditions, $t(94) = 1.08$, $p = .283$, 95% CI [-2.34, 7.89].

For sociability ratings, a significant difference was found between the two conditions, where users in the 3-Random (*M* = 69.77, *SD* = 12.54) condition rated hosts higher on sociability than those in the S1-Hidden condition, $t(94) = 3.06$, $p = .003$, 95% CI [3.19, 14.94], $d = 0.62$.

For trustworthiness ratings, users in the 3-Random (*M* = 73.96, *SD* = 12.76) condition rated hosts significantly higher than those in the S1-Hidden condition, $t(94) = 3.63$, $p < .001$, 95% CI [4.78, 16.35], $d = 0.74$.

For credibility ratings, a significant difference was found between the two conditions. Users in the 3-Random (*M* = 77.88, *SD* = 11.73) condition rated hosts higher on credibility than those in the S1-Hidden condition, $t(94) = 3.86$, $p < .001$, 95% CI [5.09, 15.91], $d = 0.78$.

Random vs Visible. When comparing the 3-Random condition data with that of Study 1's Visible condition, containing all elements of TRI, no significant differences are found on any of the measured DVs (all *t*s ≤ 1.36, *p*s > .176).

Avoided vs Hidden. Comparing the 3-Avoided triplet profile data with the S1-Hidden data revealed the same pattern of results as with the 3-Random comparisons.

Users made significantly more rent decisions in the 3-Avoided condition (*M* = 7.73, *SD* = 2.79) than in the S1-Hidden condition, $t(105) = 2.36$, $p = .20$, 95% CI [.20, 2.31], $d = 0.48$. While, again, no difference in confidence ratings were uncovered, $t < 1$, ns.

For host ratings, a significant difference was found for sociability, trustworthiness,

and credibility as a result of the profile condition. Users rated hosts higher on sociability in the 3-Avoided condition (*M* = 70.30, *SD* = 15.43) than in the S1-Hidden condition, *t*(105) = 2.36, *p* = .003, 95% CI [3.33, 15.87], *d* = 0.60. They also rated hosts higher on trustworthiness in the 3-Avoided condition (*M* = 73.94, *SD* = 16.07), *t*(105) = 3.31, *p* = .001, 95% CI [4.22, 16.86], *d* = 0.66. And, rated hosts higher on perceived credibility (3-Avoided, *M* = 75.93, *SD* = 16.02, *t*(105) = 2.74, *p* = .007, 95% CI [2.36, 14.74], *d* = 0.55.

Avoid vs Visible. As expected, when comparing the 3-Avoided condition responses with those of the S1-Visible, no significant differences were uncovered (all *t*s > .80, *p*s > .423). This suggests that three cues, even those users tend to not selected when given the opportunity, result in the same increased ratings towards hosts and decisions to rent.

Random vs Reveal S1 and S2. To ensure that the act of selecting information did not have an additional impact on user judgements beyond simply seeing three elements, comparison analyses were conducted between the 3-Random condition and the S1-Reveal condition data. Once more, the results did not indicate any significant differences in user responses, on any measure, all *t*s > .642, *p*s > .523; the same pattern was observed for the S2-3-Reveal condition comparison, *t*s > 1.114, *p*s > .268.

Avoided vs Reveal S1 and S2. Finally, the data from the 3-Avoided condition was similarly compared to that of Study 1's Reveal condition. This also did not produce any significant differences in users judgements between seeing a triplet with elements users tend to avoid selecting when making their rental decisions with those they select themselves, on any measure (S1-Reveal, all *t*s > .620, *p*s > .537; S2-3-Reveal, all *t*s > .937, *p*s > .350).

Conclusion

Thus, even when compared with the previous data where users had minimal (S1-Hidden) or full data (S1-Visible), the identical pattern of results was obtained as in Study 1 and 2. Overall, the data strongly supports the prediction that three elements of TRI are

sufficient to affect user judgement relating to hosts and rental decision.
