# Supplementary material for: Digital Identity: The effect of trust and reputation information on user judgement in the Sharing Economy
Source: PLoS One. 2018 Dec 13;13(12):e0209071. doi: 10.1371/journal.pone.0209071 (PMC6292641; doi:10.1371/journal.pone.0209071)
Supplement: S7 Text — Explanation of the data processing used for the three reported studies and description of variables in the datasets. (PDF) [file pone.0209071.s007.pdf]

Data Processing ReadMe

1. The responses provided by each participant (user) were recorded by the Gorilla platform (http://gorilla.sc/), on which our artificial SE platform was designed. This data was then exported as a CSV file containing all responses from all users. The data included demographics, judgments made on each profile, and other relevant metrics. See screengrab below for how the data files appear.

	A	C	G	H	I	J	O	R	T	V	W	X	Y	Z	AA	AB	AC	AD	AE
1	Event Inde	Date	Local Date	Experiment	Experiment	Tree Node	Participant	Task Name	Region	Rent	Validate Qu	Validate Re	Practice	Confidence	Social	GTrust	Credible	CogTrust	AffTrust
2	1	25/06/2017 11:32	25/06/2017 07:32	1164	16	task-my48	obrrmb	Reveal		1			1	8	7	7	7	8	8
3	2	25/06/2017 11:32	25/06/2017 07:32	1164	16	task-my48	obrrmb	Reveal			What colou	white	1						
4	3	25/06/2017 11:32	25/06/2017 07:32	1164	16	task-my48	obrrmb	Reveal	reputation										
5	4	25/06/2017 11:32	25/06/2017 07:32	1164	16	task-my48	obrrmb	Reveal	number-of-reviews										
6	5	25/06/2017 11:32	25/06/2017 07:32	1164	16	task-my48	obrrmb	Reveal	star-rating										
7	6	25/06/2017 11:33	25/06/2017 07:33	1164	16	task-my48	obrrmb	Reveal		0				9	2	3	3	3	3
8	7	25/06/2017 11:33	25/06/2017 07:33	1164	16	task-my48	obrrmb	Reveal			Was the ho	female							
9	8	25/06/2017 11:33	25/06/2017 07:33	1164	16	task-my48	obrrmb	Reveal	host-verification										
10	9	25/06/2017 11:33	25/06/2017 07:33	1164	16	task-my48	obrrmb	Reveal	guest-reviews										
11	10	25/06/2017 11:33	25/06/2017 07:33	1164	16	task-my48	obrrmb	Reveal	social-media										

Figure 1. Raw data from Gorilla. Each row represents one response provided by the user (e.g., ratings for the profile, element selected using their tokens, or responses to the validation question).

2. The raw data from Gorilla is then processed in Excel to arrange for data input in SPSS. Here, incomplete or incorrect responses are identified and eliminated (see main text and SI for additional details). The data for each user is also transposed to reflect a single line entry per profile. See screengrab below for how the final processed Excel file appears.

	A	B	C	D	E	F	G	H	I	J	K	L	M	N	O	P	Q	R	S	T	U	V	W	X
1	ID	Trial	User	Condition	Gender	Time	Age	Ethnicity	IC	SE_Membe	SE_use_len	SE_use_fre	SE_satisact	SE_belong	Rent	Confidence	Social	GTrust	Credible	CogTrust	AffTrust	Token1	Token2	Token3
821	42	10	ztjswb	Hidden	2	00:06:36	27	5	45	2	4	5	8	10	1	6	7	9	8	9	8	0	0	0
822	83	1	afncdm	Revealable	1	00:13:23	25	5	41	2	4	2	9	7	0	7	4	8	8	7	6	star-rating	number-of	host-verific
823	83	2	afncdm	Revealable	1	00:13:23	25	5	41	2	4	2	9	7	1	8	8	8	9	8	8	number-of	star-rating	host-verific
824	83	3	afncdm	Revealable	1	00:13:23	25	5	41	2	4	2	9	7	1	8	7	8	9	9	8	star-rating	number-of	host-verific
825	83	4	afncdm	Revealable	1	00:13:23	25	5	41	2	4	2	9	7	1	6	7	7	7	8	7	star-rating	number-of	host-verific
826	83	5	afncdm	Revealable	1	00:13:23	25	5	41	2	4	2	9	7	1	8	8	8	9	9	9	star-rating	number-of	host-verific
827	83	6	afncdm	Revealable	1	00:13:23	25	5	41	2	4	2	9	7	0	5	7	7	8	8	8	star-rating	number-of	host-verific
828	83	7	afncdm	Revealable	1	00:13:23	25	5	41	2	4	2	9	7	0	7	5	7	7	7	8	star-rating	number-of	host-verific
829	83	8	afncdm	Revealable	1	00:13:23	25	5	41	2	4	2	9	7	0	6	5	7	4	7	7	star-rating	number-of	host-verific
830	83	9	afncdm	Revealable	1	00:13:23	25	5	41	2	4	2	9	7	1	7	10	8	8	7	10	star-rating	number-of	guest-revie
831	83	10	afncdm	Revealable	1	00:13:23	25	5	41	2	4	2	9	7	1	7	6	8	9	9	9	star-rating	number-of	guest-revie

Figure 2. Excel file with user responses per each experimental condition. Each row represents the user's responses to a single profile. For this particular study, each user has 10 rows reflecting the 10 profiles they rated.

3. The Excel data is then aggregated per each user to form the final responses used in the data analysis process. In Study 1, for instance, each experimental condition had 10 profiles (as

seen in Figure 2). Summing the ratings across all 10 profiles provides the responses of that particular user in their respective experimental condition. These data are then inputted into SPSS. See screengrab below for how this appeared.

	id	user	condition	gender	time	age	ethnicity	SE_member	SE_use_length	SE_use_freq	SE_satisfaction	SE_sense_belong	IC	Rent_tot	Confidence	Social	G_trust	Credible
1	1	abvinq	Hidden	female	0:11:13	35	White	1-2	2-6 months	0-5	7	3	26.00	8.00	77.00	60.00	56.00	63.00
2	2	axutjk	Hidden	female	0:10:04	29	White	1-2	>3 years	0-5	8	3	6.00	10.00	75.00	66.00	75.00	76.00
3	3	bdshrm	Hidden	male	0:19:23	42	White	1-2	2-6 months	0-5	5	5	21.00	8.00	87.00	78.00	79.00	81.00
4	4	binotd	Hidden	female	0:14:43	25	White	1-2	12 months	5-10	8	3	30.00	5.00	76.00	53.00	57.00	76.00
5	5	bnsyfh	Hidden	female	0:15:13	25	White	3-4	>3 years	5-10	8	5	21.00	9.00	72.00	83.00	72.00	87.00

Figure 3. Example SPSS file with the final data for each Study. Each row represents a single user's judgments across the experiment, for their respective condition. The corresponding demographics for that user are also included as well as other relevant metrics.

4. The data was then analyzed in SPSS using the appropriate techniques for our hypotheses and experimental design (see main text and SI for details).
5. The final database files (one for each study) are attached with our submission and have been made available. For anyone wishing to replicate our results, simply copy/open the relevant SAV file into SPSS's workspace and refer to the SPSS manual for instructions to carry out our chosen manipulations on the data. For example, the first reported analysis in the main text is a one-way ANOVA, where Profile (Hidden, Reveal, or Visible) – named "condition" in the SAV file – is entered as the between-subjects factor of the analysis and Rent decision – named "Rent_tot" in the SAV file – is entered as the dependent measure. Link to SPSS Manual:

   https://www.ibm.com/support/knowledgecenter/SSLVMB_24.0.0/spss/product_landing.html

   Additionally, King (2013), 'Discovering Statistics using IBM SPSS Statistics' can provide additional help to SPSS users.

   Note: the user names in the included screengrabs are randomly generated validation codes by Gorilla to keep track of the data, and do not reflect any identifiable information of real people. All data is kept secure and anonymized as per the University's and EU's regulations.

6. SAV files variable descriptions:

Variable	Meaning
id	a unique number per each row to keep track of the data order
user/code	a unique code assigned to each user acting as a redundant variable for "id"
condition	reflects the experimental profile condition into which the user was randomly assigned at the start of the experiment
Super_Cond	reflects the primary experimental profile conditions, nesting any sub-conditions under one label
gender	the self-reported gender of the user
time	time taken to complete the experiment
age	the self-reported age of the user
ethnicity	the self-reported ethnicity of the user
SE_member	the number of SE platform memberships the user had
SE_use_length	the length of time a user has been using SE platforms
SE_use_freq	the reported frequency with which a user uses SE platforms
SE_satisfaction	the self-reported level of satisfaction a user has had on SE platforms
SE_sense_belong	the self-reported sense of belonging to a community derived from their participation in SE platforms
IC	the overall score on a the culture related questions of the demographics which reflect if a user has a more collectivist or individualist mentality
Rent_tot	the total number of rental decisions made by the user based on the total number of trials (i.e. profiles seen)
Confidence	the aggregate confidence in the rental decisions made by the user over the total number of trials (i.e. profiles seen)
Social	the aggregate perceived sociability of the hosts over the total number of trials
G_trust	the aggregate perceived trustworthiness of the hosts over the total number of trials
Credible	the aggregate perceived credibility of the information provided by hosts over the total number of trials
CogTrust	the aggregate perceived cognitive trustworthiness of the hosts over the total number of trials
AffTrust	the aggregate perceived affective trustworthiness of the hosts over the total number of trials
Source1	the primary source used in making a decisions on SE platforms
Source2	the secondary source used in making a decisions on SE platforms
Source3	the tertiary source used in making a decisions on SE platforms
NoSource_Reason	if no available option was selected, the user could type in a source of information that they usually rely on to make SE decisions
MoreCrossPlat	the desire to see more cross-platform information on SE platforms
Desired_info	any notes the user considered regarding information they may wish to have on SE platforms
